# Comparative analysis of canine mesenchymal stem cells and bone marrow-derived mononuclear cells

**DOI:** 10.14202/vetworld.2021.1028-1037

**Published:** 2021-04-29

**Authors:** Noritaka Maeta, Katsutoshi Tamura, Fuuna Ezuka, Hiroshi Takemitsu

**Affiliations:** 1 Aikouishida Animal Hospital, Isehara, 1195-4 Takamori, Isehara, Kanagawa, 259-1114, Japan; 2 Faculty of Veterinary Medicine, Okayama University of Science, 1-3 Ikoinooka, Imabari, Ehime, 794-8555, Japan; 3 Science and Humanities Master’s Programme, Graduate School of Science and the Humanities, Kurashiki University of Science and The Arts, 2640 Nishinoura Tsurajima Kurashiki Okayama, 712-8505, Japan; 4 Department of Comparative Animal Science, College of Life Science, Kurashiki University of Science and The Arts, 2640 Nishinoura Tsurajima Kurashiki Okayama, 712-8505, Japan

**Keywords:** bone marrow-derived mononuclear cell, canine, growth factor, interleukin, mesenchymal stem cell, secretion

## Abstract

**Background and aim:**

Mesenchymal stem cells (MSCs), which have multi-lineage differentiation potentials, are a promising source for regenerative medicine. However, the focus of study of MSCs is shifting from the characterization of the differentiation potential to their secretion potential for cell transplantation. Tissue regeneration and the attenuation of immune responses are thought to be affected by the secretion of multiple growth factors and cytokines by MSCs. However, the secretion potential of MSCs profiling remains incompletely characterized. In this study, we focused on the secretion ability related and protein mRNA expression of dog adipose tissue-derived MSCs (AT-MSC), bone marrow (BM)-derived MSCs, and BM-derived mononuclear cells (BM-MNC).

**Materials and Methods:**

Real-time polymerase chain reaction analyses revealed mRNA expression of nine growth factors and seven interleukins in these types of cells and three growth factors protein expression were determined using Enzyme-linked immunosorbent assay.

**Results:**

For the BM-MNC growth factors, the mRNA expression of transforming growth factor-β (TGF-β) was the highest. For the BM-derived MSC (BM-MSC) and AT-MSC growth factors, the mRNA expression of vascular endothelial growth factor (VEGF) was highest. BM-MSCs and AT-MSCs showed similar expression profiles. In contrast, BM-MNCs showed unique expression profiles for hepatocyte growth factor and epidermal growth factor. The three types of cells showed a similar expression of TGF-β.

**Conclusion:**

We conclude that expression of cytokine proteins and mRNAs suggests involvement in tissue repair and protection.

## Introduction

Mesenchymal stem cells (MSCs) are known to have multi-lineage differentiation potential, ability for rapid proliferation, and capacity for self-renewal [1-3]. In addition, they are widely present in tissues, such as bone marrow (BM), adipose tissue (AT), umbilical cord, and muscle [4-6]. MSCs can differentiate into various tissues in the appropriate microenvironment [7]. MSCs hold promise for regenerative medicine due to their accessibility, expandability, and multipotentiality. Recent research also finds interest in MSCs’ secretion potential, as well as their differentiation, proliferation, and self-renewal potentials [8]. Moreover, studies reveal that MSCs secrete various cytokines, such as epidermal growth factor (EGF), fibroblast growth factor (FGF), and vascular endothelial growth factor (VEGF) [9,10]. These cytokines are thought to work synergistically with the differentiation potential of MSCs in tissues repair [11,12]. Significant research also reveals the potential of MSCs to secrete immunosuppressive cytokines and their involvement in preventing acute inflammation in tissues, such as myocardium, nerves, and dacryoadenitis [12-14]. It has been suggested that these effects are caused by cytokines.

BM-MNCs have been used to treat leukemia by cell transplantation as this method is very safe [15]. Since BM-MNCs consist of MSCs, hematopoietic stem cells, fibroblasts, and other cells, they have been conjectured to have a secretion potential which is different from that of BM-MSCs. BM-MNCs are also known to be mobilized by response to the release of inflammatory cytokines in the peripheral blood by injured tissues; these cells eventually reach injured tissue and contribute to their regeneration [16]. This behavior is evident in several tissues in response to injuries, such as acute myocardial infarction, hepatic failure, and renal failure [15,17]. Moreover, we have recently performed a clinical trial that used BM-MNCs in a dog with an acute spinal cord injury resulting from intervertebral disc herniation; results showed that BM-MNC transplantation resulted in a significant increase in the recovery rate of spinal cord injury [17]. Hence, the secretion of growth factors and cytokines by BM-MNCs allowed for these recoveries.

However, there have been few studies on the secretion potential of canine BM-MNCs. In general, growth factors are proteins that promote growth and proliferation of particular cells. Brain-derived neurotrophic factor (BDNF), nerve growth factor (NGF), and neurotrophin 3 were involved in protection, proliferation, or differentiation of nerve [18,19]. EGF, FGF-2a, and hepatocyte grows factor (HGF) are related to the proliferation of many cells [20-22]. Platelet-derived growth factor-C (PDGF-C) and VEGF-A play a crucial role in angiogenesis [23] and transforming growth factor (TGF-β1) is an inducer of fibroblasts [24]. These capabilities are a function indispensable for tissue repair. Interleukin (IL) is a cytokine related to inflammation and (IL)-1a, IL-1b, IL-6, IL-11, and IL-17A are well known to promote inflammation [25-27]. However, IL-4 and IL-10 function differently for these ILs suppress inflammation [28]. Few studies have been conducted on these cytokines in an attempt to measure them simultaneously in the MSCs of the dog. Here, we evaluated the mRNA expression profile of nine growth factors, seven ILs, and TNF-α in canine BM-MSCs, AT-MSCs, and BM-MNCs by quantitative real-time polymerase chain reaction (qPCR). In addition, we examined the protein expression of VEGF-A, HGF, and TGF-β1.

This study was aimed at characterizing the secretion profile of canine MSCs and MNCs isolated from BM and AT. Our study is able to provide important findings for the basic research of cell transplantation therapy.

## Materials and Methods

### Ethical approval

Animal experiments were carried out in accordance with the National Institutes of Health guidelines for the care and use of laboratory animals. This study and the protocol included herewith were approved by the Aikouishida Animal Hospital Committee for Animal Experimentation.

### Study period and location

The study was conducted from April 2019 to November 2019 in Aikouishida Animal Hospital and Kurashiki University of Science and The Arts.

### Animals

The study included three young healthy female beagle dogs (1 year old, 9.5-11.3 kg body weight). Before tissue sample isolation, anesthesia with propofol (Hospira, Osaka, Japan) (7 mg/kg) by intravenous injection was applied. After intubation, anesthesia was maintained with isoflurane (1.5-2.0%) in oxygen.

### Cell isolation and culture

BM was aspirated from the proximal humerus with a general BM biopsy technique under anesthesia. In brief, a sterilized 13-gauge Jamshidi needle (Cardinal Health, McGaw Park, IL, USA) was used to aspirate 5 mL of BM into a syringe containing 5 mL of heparinized (1000 units/mL) saline solution. Ten ­milliliters of BM/saline mixture was carefully layered on 5 mL of density gradient medium (density, 1.077; Lymphoprep; Nycomed Pharma, Oslo, Norway) and then centrifuged at 500×*g* for 30 min. The suspended cloud-like layer of BM-MNCs was carefully collected, washed twice in 10 mL of physiological saline and centrifuged at 300×*g* for 5 min. These cells were used for the qPCR assay, and the remaining cells were separated into 6-well plates and 75-cm^2^ cell-culture flasks to separate BM-MNCs and BM-MSCs. Each cell was then resuspended in Dulbecco’s modified Eagle’s medium (Invitrogen, Carlsbad, CA, USA) with 10% fetal bovine serum (HyClone Laboratories, Utah, USA) and a 1% antibiotic–antimycotic solution (Invitrogen) and incubated at 37°C in a humidified incubator with 5% CO_2_. The 6-well-plate-seeded cells were plated at a density of 5.0 × 10^3^ cells/cm^2^ and the conditioned medium for ELISA was collected once every 2 days.

The 75-cm^2^-cell-culture-flask-seeded cells were cultured until 70-80% confluency, and then the attached cells were passaged by exposure to 0.25% trypsin for 3 min and replated at a density of 8.0×10^3^ cells/cm^2^ for subsequent passage. Passage 2 cells were separated into 6-well plates at a density of 5.0×10^3^ cells/cm^2^ for ELISA and 75-cm^2^ cell-culture flasks for qPCR.

AT was also harvested from each dog under general anesthesia. Subcutaneous fat pads (approximately 1.0 g) were harvested from the inguinal area. These pads were finely minced with scissors, digested in 40 mL of phosphate-buffered saline (PBS) containing 0.15% type-1 collagenase (Sigma-Aldrich, St. Louis, MO, USA), and then shaken vigorously at 37°C for 60 min. Subsequently, the samples were then filtered using 100 mm cell strainers (BD Biosciences, Franklin Lakes, USA) and washed with PBS. The samples were then filtered with 100-mm cell strainers (BD Biosciences,) and washed with PBS. The obtained cells were seeded into 75-cm^2^ cell-culture flasks with 10 mL of control medium and incubated in the same manner as the BM cells.

### Reverse transcription and qPCR

Total RNA was obtained from cultured BM-MSCs and AT-MSCs in passage 1. Total RNA was extracted with a TRIzol reagent (Invitrogen) according to the manufacturer’s protocol. Total RNA was measured by spectrophotometry. Total RNA (1 μg) was reverse transcribed at 42°C for 15 min in 20 μL of QuantiTect (Qiagen, Düsseldorf, Germany) after inactivation of reverse transcription by heating at 95°C for 3 min.

Previously, we conducted experiments on and mRNA expression in cultured cells [29]. The cDNA product was subjected to qPCR according to the user instructions for the Real-Time PCR System 7300 (Applied Biosystems, Foster City, CA, USA). qPCR was performed at 95°C for 5 s and 60°C for 34 s in 20 μL of buffer containing SYBR premix ExTaq II and ROX Reference Dye (Takara Bio, Shiga, Japan) and 0.2 μM each of the primers (Tables-1 and 2). Each primer was designed based on information on GenBank. The value of mRNA expression was calculated and expressed as copies (copies/ng of cDNA input) or normalized to beta-actin. Each primer is used after confirming that the PCR product has the correct sequence. A linear amplification curve from serial dilutions of plasmid DNA containing each cDNA was established through quantitative measurements. Each reaction was performed in triplicate.

**Table 1:** Primers used for qPCR.

Name	5’–3’	Direction	Position	GenBank No.
Bdnf-1	AATCCCATGGGTTACACGAA	sense	581	NM_001002975.1
Bdnf-2	GCCAGCCAATTCTCTTTTTG	anti-sense	707	NM_001002975.1
Egf-1	GCTGTGTCATTGGATGTGCT	sense	616	NM_001003094.1
Egf-2	CTGATTCCCAAAAAGGGACAT	anti-sense	780	NM_001003094.1
Fgf-1	CACTTCAAGGACCCCAAGAG	sense	190	XM_003432481.1
Fgf-2	ACAACGCCTCTCTCTTCTGC	anti-sense	332	XM_003432481.1
Hgf-1	ATGGGGAATGAGAAATGCAG	sense	1915	AB090353.1
Hgf-2	AAAAATGCCAGGACGATTTG	anti-sense	2124	AB090353.1
Ngf-1	GTGCTGGGAGAGGTGAACAT	sense	475	NM_001194950.1
Ngf-2	GGTGGTGGTGCAGTAGGAGT	anti-sense	612	NM_001194950.1
Nt3-1	TGGCATCCAAGGTAACAACA	sense	311	XM_003433475.1
Nt3-2	GCAGGGTGCTCTGGTAGTTC	anti-sense	459	XM_003433475.1
Pdgf-1	TCTTGGCAAGGCTTTTGTTT	sense	849	XM_539783.3
Pdgf-2	TTCCCTTATGGACACCGAGA	anti-sense	966	XM_539783.3
Tgf-1	GGCCCTGGACACCAACTACT	sense	891	NM_001003309.1
Tgf-2	GCTCATGGATCCACTTCCAG	anti-sense	997	NM_001003309.1
Vegf-1	CTACCTCCACCATGCCAAGT	sense	325	NM_001003175.2
Vegf-2	AGATGTCCACCAGGGTCTCA	anti-sense	458	NM_001003175.2

qPCR=Quantitative real-time polymerase chain reaction

**Table 2:** Primers used for qPCR.

Name	5’- 3’	Direction	Position	GenBank No.
IL-1a-1	TTGTGAGTGCCCAAAATGAA	sense	648	NM_001003157.2
IL-1a-2	CCTGTGTGGCAATGAACAAC	anti-sense	812	NM_001003157.2
IL-1b-1	AGTTGCAAGTCTCCCACCAG	sense	149	NM_001037971.1
IL-1b-2	TATCCGCATCTGTTTTGCAG	anti-sense	325	NM_001037971.1
IL-4-1	CTCACCTCCCAACTGATTCC	sense	70	NM_001003159.1
IL-4-2	AGTCGTTTCTCGCTGTGAGG	anti-sense	202	NM_001003159.1
IL-6-1	GGCTACTGCTTTCCCTACCC	sense	108	U12234.1
IL-6-2	TTTTCTGCCAGTGCCTCTTT	anti-sense	305	U12234.1
IL-10-1	AGAACCACGACCCAGACATC	sense	300	U33843.1
IL-10-2	CCGCCTTGCTCTTATTCTCA	anti-sense	425	U33843.1
IL-11-1	CGGCTGGAAATTTGTCTCTC	sense	251	XM_848962.2
IL-11-2	GGCCAGATAGAGCTGCTG	anti-sense	456	XM_848962.2
IL-17-1	CCGATCTACCTCACCTTGGA	sense	214	NM_001165878.1
IL-17-2	TCGCAGAACCAGGATCTCTT	anti-sense	379	NM_001165878.1
Tnf-1	ACCACACTCTTCTGCCTGCT	sense	133	NM_001003244.4
Tnf-2	CTTGGGGTTCGAGAAGATGA	anti-sense	254	NM_001003244.4
β-actin	GCCAACCGTGAGAAGATGACT	sense	339	AF021873
β-actin	CCCAGAGTCCATGACAATACCAG	anti-sense	446	AF021873

qPCR=Quantitative real-time polymerase chain reaction

### Enzyme-linked immunosorbent assay (ELISA)

Previously, we conducted experiments on protein expression in cultured cells [29]. The samples used for the experiments were obtained from BM-MNC, BM-MSC, and Adipose-derived-MSC (AD-MSC) culture-conditioned medium and collected and frozen at −80°C until the assay. HGF (Hepatocyte growth factor ELISA kit; USCN, Huston USA), VEGF-A (Quantikine ELISA canine VEGF; R&D Systems, Inc., Minneapolis, MN, USA), and TGF-β1 (Quantikine ELISA Mouse/Rat/Porcine/Canine TGF-β1; R&D Systems, Inc.) were determined according to the manufacturer’s protocol. D-MEM containing 10% FBS was used as a blank. All ELISA kits were guaranteed by the manufacturer for use in dogs.

### Statistical analysis

Experimental values are expressed as mean ± standard error of the mean. A Holm–Sidak 1-way analysis of variance (ANOVA) was utilized to perform multiple group comparisons. Statistical significance was set at p<0.05 for both the U-test and the one-way ANOVA. The statistical analyses were performed using Prism software (GraphPad Software Inc., San Diego, CA, USA).

## Results

### Growth factor mRNA expression profiles in BM-MNCs, BM-MSCs, and AT-MSCs

**Figure-1:**
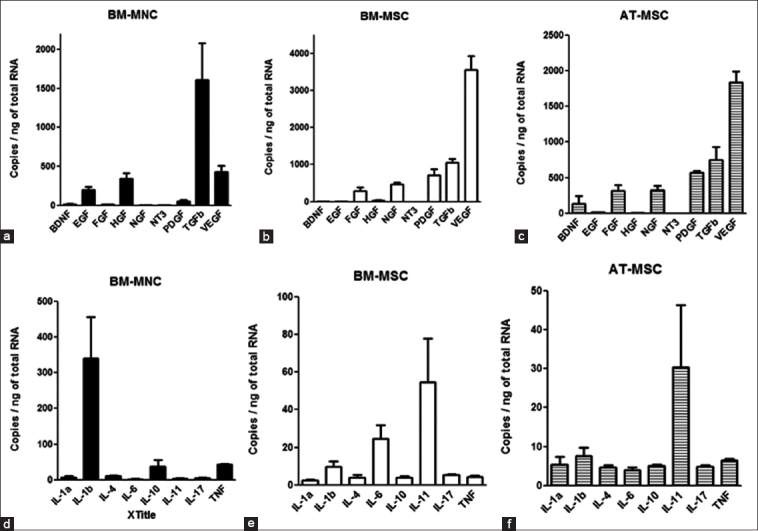
Quantitative real-time polymerase chain reaction. The levels of expression of the dog cytokine mRNAs in bone marrow-derived mononuclear cells, adipose tissue-derived mesenchymal stem cells, and bone marrow-derived mesenchymal stem cells. Each value of mRNA expression was calculated and expressed as copies (copies/ng of cDNA input). Means±SE, n=3.

Total RNA was obtained from cultured BM-MSCs and AT-MSCs in passage 2. BM-MNCs were obtained by isolation from BM immediately after separation with Lymphoprep. qPCR was used to examine the expression levels of the growth factors in BM-MNCs, BM-MSCs, and AD-MSCs (Figure-1). For the BM-MNC growth factors, the expression of TGF-β1 was highest. A moderate level of expression in BM-MNCs was observed for VEGF-A, HGF, and EGF. Furthermore, a low level of expression in BM-MNCs was observed for PDGF-C, BDNF, FGF-2a, and NGF (Figure-1a).

For the BM-MSC growth factors, the expression of VEGF-A was highest. A moderate level of expression in BM-MSCs was observed for TGF-β1, PDGF-C, NGF, and FGF-2a. Moreover, a low level of expression in BM-MSCs was observed for HGF, BDNF, EGF, and NT-3 (Figure-1b).

For the AT-MSC growth factors, the expression of VEGF-A was highest. A moderate level of expression in AT-MSCs was observed for TGF-β1, PDGF-C, NGF, FGF-2a, and BDNF. Moreover, a low level of expression in AT-MSCs was observed for EGF, HGF, and NT-3. Each value of mRNA expression was calculated and expressed as copies (Figure-1c).

### ILs and TNF-α mRNA expression profiles in BM-MNCs, BM-MSCs, and AT-MSCs

For the ILs and TNF-α in BM-MNCs, the expression of IL-1b was highest. A moderate level of expression in BM-MNCs was observed for TNF-α, IL-10, and IL-4. A low level of expression in BM-MNCs was observed for IL-17A, IL-1a, IL-11, and IL-6 (Figure-1d). For the ILs and TNF-α in BM-MSCs, the expression of IL-11 was highest. A moderate level of expression in BM-MSCs was observed for IL-6. A low level of expression in BM-MSCs was observed for IL-1b, IL-17A, TNF-a, IL-10, IL-4, and IL-1a (Figure-1e). For the ILs and TNF-α in AT-MSCs, the expression of IL-11 was highest. A low level of expression in AT-MSCs was observed for IL-1b, TNF-α, IL-1a, IL-10, IL-17A, IL-4, and IL-6 (Figure-1f). Each value of mRNA expression was calculated and expressed as copies (copies/ng of total RNA).

### Growth factor mRNA expression

**Figure-2:**
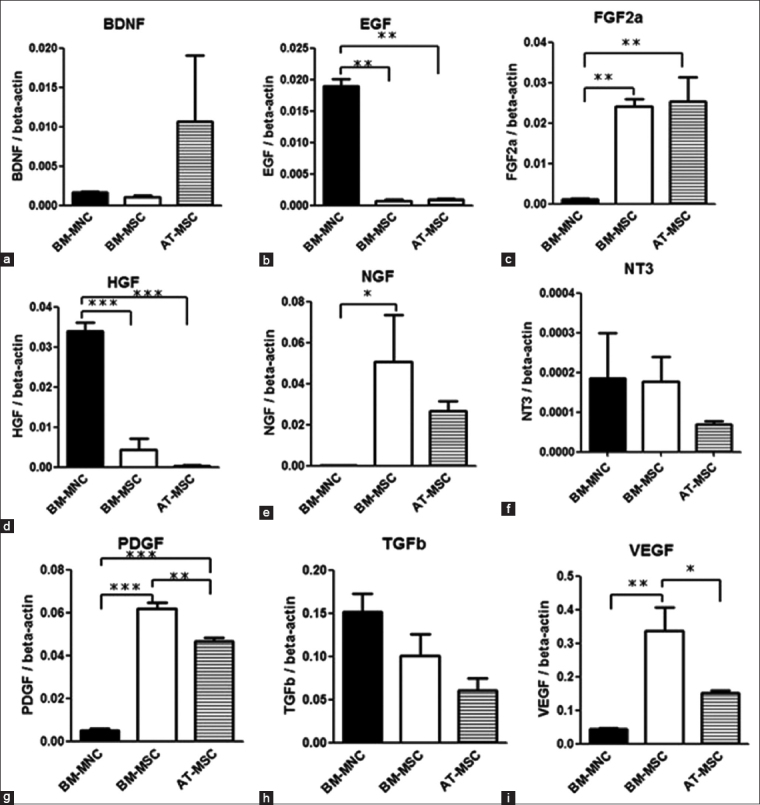
Quantitative real-time polymerase chain reaction. The levels of expression of dog growth factors mRNAs compared with those of bone marrow-derived mononuclear cells, adipose-derived-mesenchymal stem cells, and bone marrow-derived mesenchymal stem cells. Each value was normalized to beta-actin expression. Statistical comparisons were made with one-way analysis of variance (ANOVA) (*p<0.05) (**p<0.01) (***p<0.001). Means±SE, n=3.

The mRNA expression of BM-MSC and AT-MSC growth factors tended to be similar, with the exception of a few growth factors, such as BDNF, PDGF-C, and VEGF-A (Figure-2 and Table-3). However, a significant difference between BM-MNC expression compared with BM-MSCs and AT-MSCs was observed. EGF expression in BM-MNCs was 22.8 fold and 23.9 fold higher than that of BM-MSCs and AT-MSCs, respectively, and HGF expression was 8.0 fold and 87.0 fold higher, respectively. Conversely, FGF-2a, NGF, PDGF-C, and VEGF-A mRNA expressions in BM-MSCs and AT-MSCs were higher than those of BM-MNCs. FGF-2a expression in BM-MSCs and AT-MSCs was 22.8 fold and 23.9 fold higher, respectively, than that in BM-MNCs and NGF expression in BM-MSCs and AT-MSCs was 240.0 fold higher than that in BM-MNCs. PDGF-C expression in BM-MSCs and AT-MSCs was 12.4 fold and 1.3 fold higher, respectively, than that in BM-MNCs and VEGF-A expression in BM-MSCs and AT-MSCs was 7.7 fold and 2.2 fold higher, respectively, than that in BM-MNCs. BDNF, NT-3, and TGF-β1 were not significantly different between BM-MNCs and BM-and AT-MSCs. Quantitative measurements were performed by establishing a linear amplification curve from serial dilutions of plasmid DNA containing each cDNA. Each value was normalized to the expression levels of beta-actin.

**Table 3:** Growth factors expression on canine BM-MNC, BM-MSC, and AD-MSC.

	BM-MNC	BM-MSC	AD-MSC
BDNF	1.6×10^−3^±0.11×10^−3^	1.0×10^−3^±0.27×10^−3^	10.0×10^−3^±8.4×10^−3^
EGF	190×10^−4^±11×10^−4^	6.8×10^−4^±2.9×10^−4^	9.7×10^−4^±1.5×10^−4^
FGF2a	1.0×10^−3^±0.29×10^−3^	24×10^−3^±1.7×10^−3^	25×10^−3^±6×10^−3^
HGF	340×10^−4^±22×10^−4^	42×10^−4^±29×10^−4^	3.9×10^−4^±1.6×10^−4^
NGF	0.2×10^−3^±0.072×10^−3^	51×10^−3^±23×10^−3^	27×10^−3^±0.49×10^−3^
NT3	1.9×10^−4^±1.1×10^−4^	1.8×10^−4^±0.61×10^−4^	0.7×10^−4^±0.07×10^−4^
PDGF-C	0.5×10^−2^±0.1×10^−2^	6.2×10^−2^±0.29×10^−2^	4.7×10^−2^±0.18×10^−2^
TGF-β1	1.5×10^−1^±0.21×10^−1^	1.0×10^−1^±0.25×10^−1^	0.61×10^−1^±0.13×10^−1^
VEGF-A	0.43×10^−1^±0.025×10^−1^	3.4×10^−1^±0.7×10^−1^	1.5×10^−1^±0.082×10^−1^

BM-MNC=Bone marrow-derived mononuclear cells, BM-MSC=Bone marrow-derived mesenchymal stem cells, AD-MSC=Adipose-derived-mesenchymal stem cells, BDNF=Brain derived neurotrophic factor, VEGF-A=Vascular endothelial growth factor-a, TGF-β1=Transforming growth factor-β, PDGF-C=Platelet derived growth factor-C, NT3=Neurotrophin 3, HGF=Hepatocyte grows factor, NGF=Nerve growth factor, FGF2a=Fibroblast growth factor, BDNF=Brain-derived neurotrophic factor, EGF=Epidermal growth factor

### ILs and TNF-α mRNA expression

**Figure-3:**
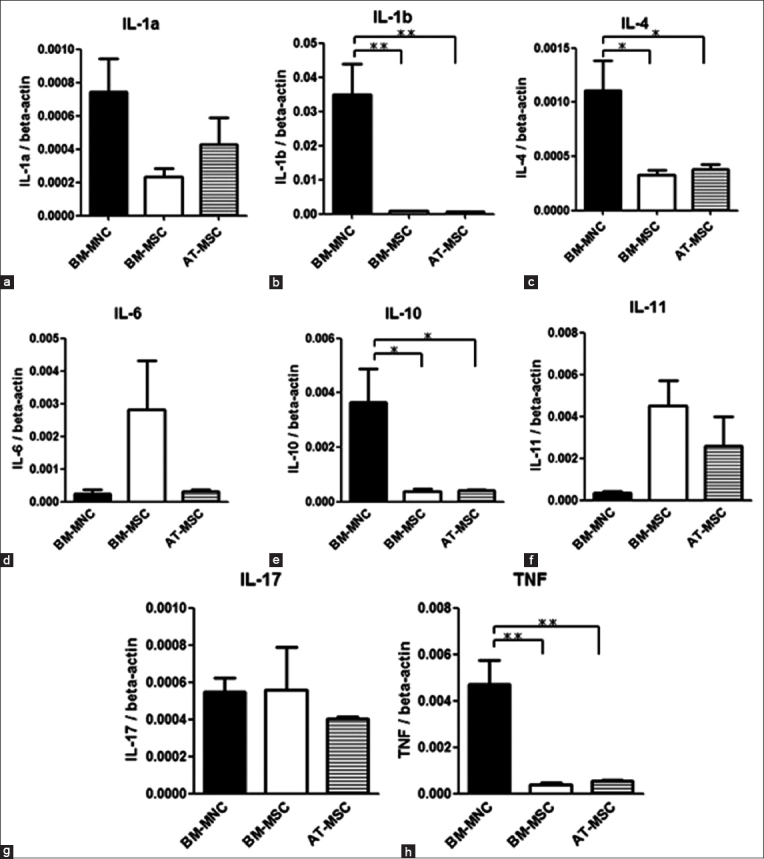
Quantitative real-time polymerase chain reaction. Expression levels of dog interleukin mRNAs compared with those of bone marrow-derived mononuclear cells, adipose-derived-mesenchymal stem cells, and bone marrow-derived mesenchymal stem cells. Each value was normalized to beta-actin expression. Statistical comparisons were made with one-way ANOVA (*p<0.05) (**p<0.01) (***p<0.001). Means±SE, n=3.

The mRNA levels of expression of ILs and TNF-α in BM-MNCs, BM-MSCs, and AT-MSCs were similar to those of growth factors (Figure-3 and Table-4). In addition, mRNA expression levels of ILs and TNF-α did not show significant differences between BM-MSCs and AT-MSCs. However, the values in BM-MNCs showed a different tendency. IL-1b expression in BM-MNCs was 41.6 fold and 57.3 fold, IL-4 was 3.5 fold and 2.9 fold; IL-10 was 9.6 fold and 8.8 fold, and TNF-α was 12.0 fold and 8.7 fold higher than those in BM-MSCs and AT-MSCs, respectively. Quantitative measurements were performed by establishing a linear amplification curve from serial dilutions of plasmid DNA containing each cDNA. Each value was normalized to the expression levels of beta-actin.

**Table 4:** Interleukins expression on canine BM-MNC, BM-MSC, and AD-MSC.

	BM-MNC	BM-MSC	AD-MSC
IL-1a	7.4×10^−4^±2.0×10^−4^	2.3×10^−4^±0.52×10^−4^	4.3×10^−4^±1.6×10^−4^
IL-1b	35×10^−3^±9.0×10^−3^	0.84×10^−3^±0.14×10^−3^	0.6×10^−3^±0.16×10^−3^
IL-4	1.1×10^−3^±0.28×10^−3^	0.32×10^−3^±0.045×10^−3^	0.38×10^−3^±0.044×10^−3^
IL-6	0.24×10^−3^±0.13×10^−3^	2.8×10^−3^±1.5×10^−3^	0.43×10^−3^±0.049×10^−3^
IL-10	3.6×10^−3^±1.3×10^−3^	0.38×10^−3^±0.095×10^−3^	0.4×10^−3^±0.049×10^−3^
IL-11	0.36×10^−3^±0.064×10^−3^	4.5×10^−3^±1.2×10^−3^	2.6×10^−3^±1.4×10^−3^
IL-17A	5.5×10^−4^±1.3×10^−4^	5.6×10^−4^±2.3×10^−4^	4.0×10^−4^±0.12×10^−4^
TNF-α	4.7×10^−3^±1.0×10^−3^	0.4×10^−3^±0.057×10^−3^	0.54×10^−3^±0.032×10^−3^

BM-MNC=Bone marrow-derived mononuclear cells, BM-MSC=Bone marrow-derived mesenchymal stem cells, AD-MSC=Adipose-derived-mesenchymal stem cells, BDNF=Brain-derived neurotrophic factor, TGF-β1=Transforming growth factor-β

### Growth factor secretion in cell culture

**Figure-4:**
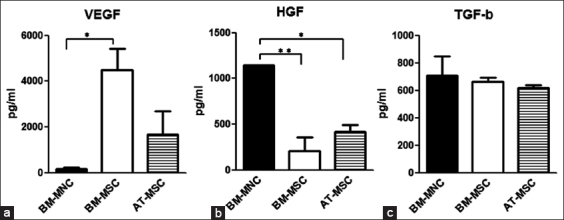
Evaluation of protein expression. The expression levels of vascular endothelial growth factor (VEGF), hepatocyte growth factor (HGF), and transforming growth factor (TGF)-β1 protein were evaluated by enzyme-linked immunosorbent assays (ELISA). Comparison of each protein expression level among bone marrow-derived mononuclear cells, adipose-derived-mesenchymal stem cells, and bone marrow-derived mesenchymal stem cells cultured conditioned medium. Statistical comparisons were made with one-way ANOVA (*p<0.05) (**p<0.01). Means±SE, n=3.

Growth factor protein was measured by selecting the growth factors with the three highest levels of mRNA expression in BM-MNCs in HGF, VEGF-A, and TGF-β1 (Figure-4). The experimental samples of each cell in a cultured-conditioned medium that was made every 2 days were used. Results showed that the levels of expression of TGF-β1 did not differ significantly between BM-MNCs, BM-MSCs, and AT-MSCs. However, VEGF-A levels of expression were higher in BM-MSCs than in BM-MNCs, whereas HGF levels of expression in BM-MNCs were higher than those in BM-MSCs and AT-MSCs (p<0.01).

### Measurement of the chronology of expressed growth factors in BM-MNCs

**Figure-5:**
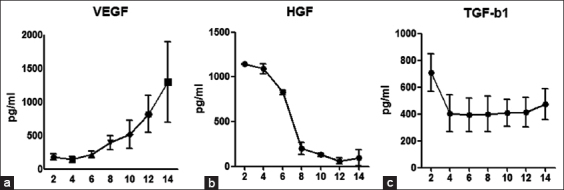
Evaluation of the chronology of the expressed growth factors in bone marrow-derived mononuclear cells. The expression levels of vascular endothelial growth factor-a (VEGF-A), hepatocyte grows factor, and transforming growth factor-β1 protein were evaluated by ELISA. Samples were collected every 2 days for 14 days. Means±SE.

To confirm the changes in the expression of HGF, VEGF-A, and TGF-β1 in BM-MNCs, we analyzed the previously conditioned medium (Figure-5). Samples were collected every 2 days for 14 days. The expression of TGF-β1 did not differ significantly between the start point (Day 2) and the endpoint (Day 14). However, a significant difference between the start point and the endpoint was found for the expression of VEGF-A and HGF: The expression of VEGF-A at the start point was very low, but it gradually increased to a stable level by 6-8 days. On the other hand, the expression of HGH at the start point was very high, but it gradually decreased to a stable level by 6-8 days.

### Evaluation of cell surface markers and differentiation potentials

This study was performed using cells that have been previously evaluated for the following cell surface markers [29]: CD29 (BM-MNC: 10.67±1.38%; BM-MSC: 98.41±0.53%; AT-MSC: 97.85±0.94%), CD34 (BM-MNC: 8.33±0.46%; BM-MSC: 0.88±0.21%; AT-MSC: 0.25±0.06%), and CD90 (BM-MNC: 25.17±0.18%; BM-MSC: 19.1±2.1%; AT-MSC: 22.55±2.8%). The differentiation potential of BM-MSCs and AT-MSCs has been confirmed previously.

## Discussion

In this study, no significant difference in the mRNA expression and secretion of cytokines and ILs between BM-MSCs and AD-MSCs was observed. However, higher expression levels of EGF, HGF, IL-1b, IL-4, IL-10, and TNF-α in BM-MNCs were observed in comparison with those in MSCs. In addition, the levels of expression of FGF-2a, NGF, PDGF-C, and VEGF-A were also higher. BM-MNCs were found to be lower than those of MSCs. Thus, the change of BM cell character by culturing was confirmed. Moreover, TGF-β1, which is a pleiotropic cytokine that regulates extracellular matrix production, cell proliferation, and differentiation, showed high levels of expression in all three cells that secreted cytokines.

Moreover, cytokines with high expression in BM-MNCs, such as EGF, HGF, and IL-10, have been predicted to be effective in the anti-inflammatory response and tissue regeneration [30-32]. EGF has the ability to activate cell division through the promotion of mitosis [32]. IL-10’s primary role is an anti-inflammatory effect that occurs by inhibiting the differentiation of inflammatory cells and the secretion of inflammatory cytokines [31]. In particular, HGF is expected to be effective in the treatment of various diseases. Studies showed that HGF has an anti-apoptotic ability in cells and has a regenerative and protective ability in various tissues [33-35]. In addition, a study revealed HGF to have anti-fibrotic and anti-inflammatory effects [36]. These effects may be due to BM-MNC transplanted [17]. On the other hand, IL-1d and TNF-α are known inflammatory cytokines to have high expression in BM-MNCs [25,36]. Therefore, cell transplantation might lead to exacerbation of temporary inflammation. Furthermore, this runs the risk of worsening symptoms, depending on the purpose of the treatment. BM-MNCs are expected to have cell proliferation, anti-apoptosis, and anti-fibrotic effects.

In contrast, AT-MSC and BM-MSC cytokine expression, such as NGF, PDGF-C, VEGF-A, and FGF-2a, was higher than that in BM-MNCs. NGF has the ability to repair neural tissue with wounds [37], whereas PDGF-C promotes cellular proliferation and inhibits apoptosis [20]. VEGF-A constitutes a family of regulatory peptides that are capable of controlling blood vessel formation and permeability [38]. FGF-2a has been related to the differentiation control of various organs, such as the heart, liver, pancreas, and bones [39]. Moreover, FGF-2a is related to angiogenesis with PDGF-C and VEGF-A. In particular, the interaction of FGF-2a and VEGF-A contributes stable angiogenesis [40]. For this reason, AT-MSCs and BM-MSCs have been considered to contribute to cell proliferation and neural repair and angiogenesis.

Our study revealed the mRNA and protein expression profiles of canine BM-MNCs, BM-MSCs, and AT-MSCs, showing BM-MSCs and AT-MSCs to have similar expression profiles. In contrast, BM-MNCs showed unique expression profiles for HGF and EGF. The three types of cells showed a similar expression of TGF-β1. The secretion ability of MNCs might be appropriate at early stages of inflammation during transplantation due to its anti-apoptotic effect. On the other hand, the secretion ability of MSCs might be appropriate during transplantation, that is, in tissue repair period, due to its cell proliferation and angiogenesis effect. However, this study only used three subjects and tendencies may be different depending on the number of samples of BDNF, NGF, IL-6, and IL-10.

## Conclusion

Changes in chronologies secretion ability of growth factors seems to reflect the results of mRNA expression. TGF-β1 secretion did not differ significantly between BM-MSC and MNC functions. In contrast, HGF secretion was decreased by time-dependent and VEGF secretion was increased by time-dependent. This result suggests that the transplantation effects obtained were different. BM-MNC was shown to have a different ability to secrete cytokines than BM-MSC and AT-MSC. And BM-MNCs were easy to handle because it does not require culturing. These suggest that BM-MNC may be applicable to various disease treatments. We found the protein and mRNA expression of cytokines that may help for tissue repair and protection, but it remains unclear whether the activity level that is required for the repair and protection of each tissue is obtained in practice. Further studies of *in vivo* and coculture experiments are required.

## Authors’ Contributions

NM and KT designed the study, drafted the manuscript, and analyzed data. FE collected samples and did ELISA analysis. HT contributed to the study design and helped in editing and revision of the manuscript. All authors read and approved the final manuscript
